# Decoding rheumatoid arthritis: Biomarker identification and immune profiling via bioinformatics and Mendelian randomization

**DOI:** 10.1097/MD.0000000000043872

**Published:** 2025-08-22

**Authors:** Shoujia Shao, Wenxing Zeng, Jingtao Zhang, Luyao Ma, Feng Huang, Ziwei Jiang

**Affiliations:** a Guangzhou University of Chinese Medicine, Guangzhou, China; b The First Affiliated Hospital of Guangzhou University of Chinese Medicine, Guangzhou, China.

**Keywords:** autoimmune diseases, biomarkers, gene expression analysis, genetic markers, immune cell infiltration, Mendelian randomization, rheumatoid arthritis, therapeutic targets

## Abstract

Rheumatoid arthritis (RA) is a prevalent autoimmune disorder that significantly reduces quality of life and imposes a substantial burden on society. This study addresses the critical gaps in current diagnostic and therapeutic modalities by aiming to identify improved biomarkers and potential therapeutic targets. Using data from 2 gene expression omnibus databases, we executed a comprehensive differential gene expression analysis integrated with Mendelian randomization. This approach employed advanced bioinformatics tools to scrutinize expression quantitative trait loci (eQTLs) and RA genome-wide association study data to pinpoint crucial genes involved in RA. The selection of these pivotal genes was strategically based on the intersection of upregulated gene expressions with eQTLs exhibiting odds ratios >1, and conversely, downregulated gene expressions aligned with eQTLs displaying odds ratios <1. Our enrichment analyses, including gene ontology, Kyoto encyclopedia of genes and genomes, and gene set enrichment analysis, provided robust validation of these genes’ roles, further supported by external validation from an additional gene expression omnibus dataset. The study identified 13 critical genes related to RA susceptibility, including CKAP2, GABBR1, HLA-DPA1, ST6GAL1, FCGR1A, ADCY7, MAP4K1, CD37, ERAP2, and SEMA3C, alongside protective genes. An in-depth analysis of immune cell infiltration underscored the dominant roles of M2 macrophages and CD8+ T cells in the RA immune microenvironment, highlighting their significant contributions to disease pathogenesis. By identifying novel biomarkers and elucidating the dynamic immune landscape of RA, our findings lay the groundwork for innovative therapeutic strategies. This study significantly advances our understanding of the complex genetic mechanisms underlying RA, offering insights that pave the way for targeted therapeutic interventions and further research into the molecular drivers of RA.

**Key points**

- Identified 13 critical genes associated with RA susceptibility and validated these findings across additional databases, confirming significance for 7 genes.
- Innovatively utilized eQTL for Mendelian randomization combined with comprehensive bioinformatics analyses in RA research.
- Conducted GO, KEGG, and GSEA enrichment analyses, revealing multiple signaling pathways involved.
- Emphasized the significant roles of M2 macrophages and CD8+ T cells in RA pathogenesis, offering profound insights into novel biomarkers and therapeutic targets for RA.

## 1. Introduction

Rheumatoid arthritis (RA) is a chronic, symmetrical autoimmune disease, characterized by its aggressiveness and frequent involvement of multiple body joints. The global prevalence is approximately 5 per 1000, with the condition being 2 to 3 times more common in women than in men.^[1]^ RA is characterized by pain, morning stiffness, joint erosion, destruction, and resultant limb deformities. Some RA patients may develop manifestations in organs beyond the joints, such as cutaneous rheumatoid nodules, pericarditis, and interstitial lung lesions, underscoring RA’s nature as a multisystem disease.^[2]^ Diagnosis is primarily based on clinical symptoms, signs, and laboratory and imaging tests, which can lead to missed diagnoses of early, atypical, or inactive RA. Recent genome-wide association study (GWAS) and meta-analyses have revealed common disease-associated variants, increasing the potential for early diagnosis and clinical treatment.^[3,4]^ However, the exact genes involved in RA pathogenesis remain unclear, necessitating further exploration for new diagnostic genes and treatment strategies.

Gene-specific expression profiling, a bioinformatics approach, has recently gained wide use in analyzing microarray data.^[5]^ Microarray technology is extensively applied to study gene expression patterns in bone and synovial tissues of RA patients or experimental animals.^[6]^ However, inconsistencies in microarray studies, including varying platforms, sample sizes, data outliers, and sources, have not been fully addressed.^[7]^ To enhance computational efficiency and statistical accuracy, our study employed the robust rank aggregation (RRA) method for integrating differential expression profiles from selected datasets.^[8]^ Previous studies on RA bioinformatics analysis did not systematically incorporate differentially expressed genes (DEGs) using the RRA method, which facilitated this study. Mendelian randomization (MR), an epidemiological method, was utilized to detect the causal effects of exposures on outcomes (RA), using genetic variants as instrumental variables (IVs). This method, avoiding environmental interference and reverse causation, has been widely used to identify causal genes.^[9]^ Recently, GWAS of expression quantitative trait loci (eQTL) have identified thousands of genes, making eQTL a valuable tool for estimating the causal impact of genes on complex diseases and aiding in drug target prioritization and repurposing.^[10]^

Therefore, our study aimed to identify key genes associated with disease onset and assess the therapeutic efficacy of RA at the genetic target level, thereby offering insights for the development of novel targeted drugs for RA. By integrating gene expression omnibus (GEO) microarray data using the RRA approach and conducting MR with eQTLGen consortium cohort data and GWAS outcomes for RA, we identified core target genes. These genes were further analyzed for biological functions and mechanisms via gene enrichment and pathway annotation. Immune cell infiltration analysis was utilized to explore significant immune cells associated with RA pathogenesis,^[11]^ and key genes were externally validated with GEO datasets to enhance result robustness. Our findings aim to facilitate the development of novel therapeutic strategies and drug targets for RA.

## 2. Materials and methods

### 2.1. Study design

**Figure 1.**
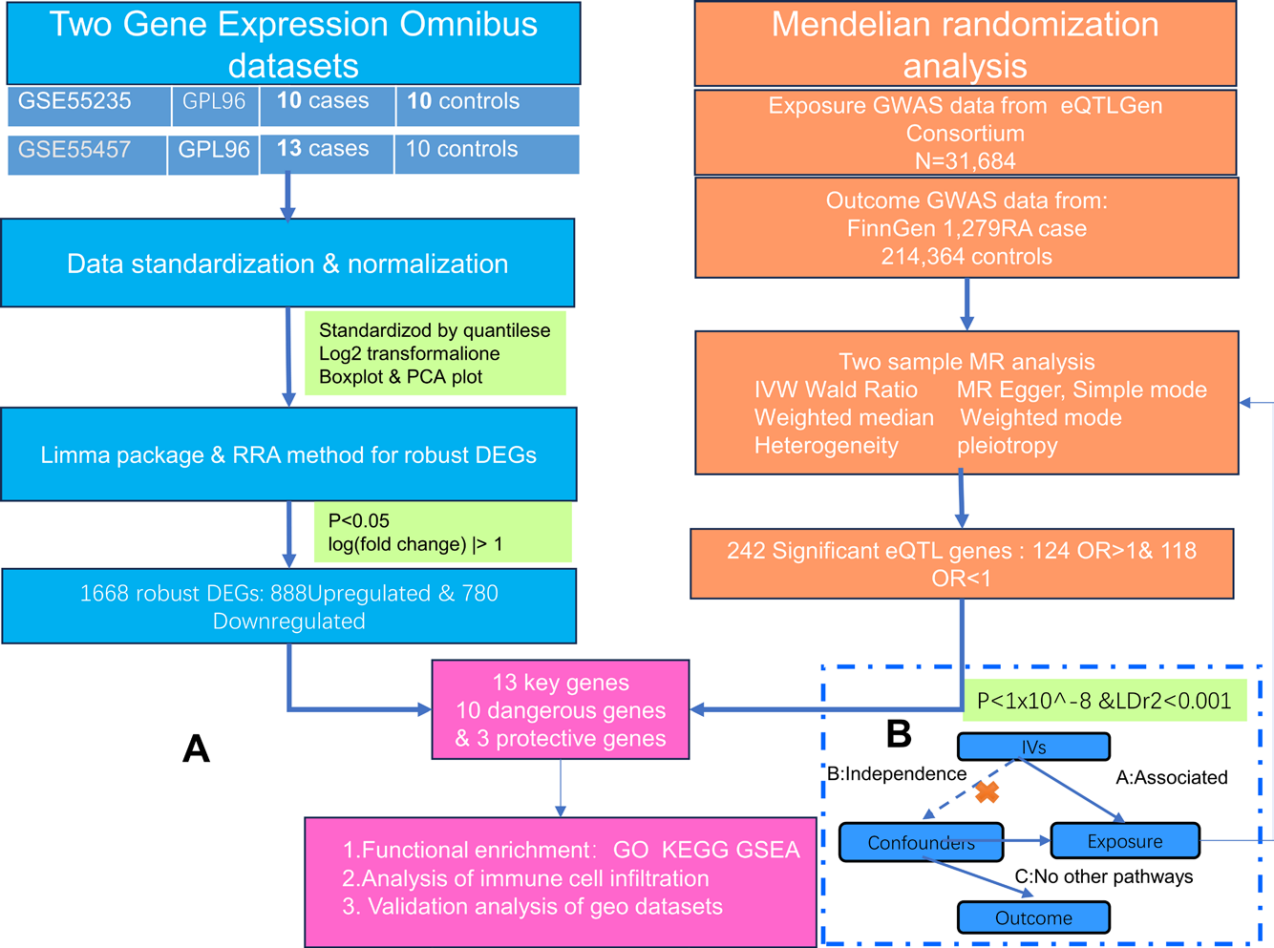
(A) Research workflow diagram. (B) MR 3 major hypotheses. MR = Mendelian randomization.

Figure 1A illustrates the research workflow. Initially, we applied the RRA method to identify DEGs from 3 GEO datasets, selecting 2 for the training group and the remaining dataset for the validation group. During this process, training datasets were amalgamated, and batch effects were mitigated. Subsequent to this, TSMR, employing data from a large-scale eQTL study and GWAS data for RA, was conducted. Figure 1B delineates the 3 principal hypotheses of MR.^[12]^ Significant eQTLs or genes causally linked to RA were identified (penalized inverse-variance weighted estimator < 0.05), and their intersection with the DEGs from the GEO database’s training set was determined (with up-regulated genes corresponding to MR odds ratio > 1), thereby identifying the key genes associated with RA. Subsequently, these key genes underwent gene ontology (GO) function and Kyoto encyclopedia of genes and genomes (KEGG) pathway enrichment analyses^[13]^ to elucidate biological mechanisms intimately associated with RA. Gene set enrichment analysis (GSEA) enrichment analysis^[14]^ was then conducted on the key genes with the most significant up- and down-regulated effect sizes, to identify pathways and biological functions of these genes, thereby analyzing their role in RA pathogenesis. Subsequently, the GEO DEGs underwent immune cell infiltration analysis, to examine significant immune cells involved in RA pathogenesis and their direct interactions with the key genes. Lastly, the identified key genes were validated using external GEO datasets.

### 2.2. RA GEO dataset

Our exploration began with a detailed search of the GEO repository, using a comprehensive set of search terms: “rheumatoid arthritis,” “rheumatism,” “gene expression,” “Homo sapiens,” and “microarray.” This inquiry aimed to identify datasets meeting rigorous selection criteria: (1) inclusion of at least 6 samples in total, with a balanced representation of at least 3 case and 3 control samples; (2) exclusion of samples subjected to chemical or genetic interventions; and (3) availability of either raw data or detailed gene expression profiles, to ensure the integrity and comparability of our analysis.

### 2.3. Standardization and integration of data

Gene expression data and annotations were retrieved from the GEO database. Probe identifiers were mapped to gene symbols for further analysis. The data underwent normalization via mean centering and log2 transformation.^[15]^ We selected GSE55235 and GSE55457 as the training set and identified intersecting genes across datasets to enable integration. Principal component analysis was utilized to visualize the primary biological expression patterns and assess batch effect removal, thereby reducing random error and improving data analysis integrity.^[16]^

### 2.4. Differential gene expression analysis via RRA

The limma package in R (version 4.2.3) was utilized to identify DEGs between RA patients and healthy controls, applying a threshold of |logFC| > 1 and *P* < .05.^[17]^ Visualization tools including R packages “ggplot2,” “ggrepel,” “limma,” and “pheatmap” were employed to generate heatmaps and volcano plots from the merged and corrected datasets, thereby visualizing differential gene expression.

### 2.5. MR statistical framework

#### 2.5.1. Source of GWAS data

eQTL data were extracted from the eQTLGen consortium, involving a comprehensive meta-analysis of 31,684 blood samples across 37 cohorts, as conducted by Vosa U et al, with all participants being of European descent.^[18]^ The RA GWAS dataset, identified as finn-b-RHEUMA_SEROPOS_STRICT, was acquired from the Finnish database, encompassing a total sample size of 215,661, including 1297 cases and 214,364 controls.^[19]^ All data were made available on the IEU OpenGWAS database (https://gwas.mrcieu.ac.uk/), where GWAS summary statistics utilized in this study are publicly accessible for download at no cost. Ethical approval was secured through the original analysis.

#### 2.5.2. IV selection

In selecting genetic instruments, 3 critical assumptions were required to be met. The first assumption (correlation) necessitated the aggregation to remove single nucleotide polymorphisms (SNPs) in linkage disequilibrium (*r*^2^ < 0.001) and selection of traits at the genome-wide significance level (*P* < 5 × 10^‐8^). The instrumental strength of each SNP was evaluated using the *F*-statistic = (β/SE)^2^, with an *F*-statistic > 10 denoting a strong instrument. This criterion was satisfied by all selected IVs, significantly reducing bias in our analysis. The second assumption (independence) posited that genetic variations should not be directly associated with any confounders, assessable by evaluating horizontal pleiotropy in post hoc MR analyses. The third assumption (exclusion) implied that IVs should influence the outcome solely through the exposure. Screening through Phenoscanner (http://www.phenoscanner.medschl.cam.ac.uk/) was performed to exclude any potential confounders, thereby minimizing confounding factors.^[20]^

#### 2.5.3. Application of MR

Upon selecting eligible IVs and filtering them with LD *r*^2^ < 0.001, datasets with fewer than 3 IVs were excluded. MR tests included the inverse variance weighted (IVW) test^[21]^ as the primary method for cases where horizontal pleiotropy was either absent or balanced, providing unbiased causal effect estimates between exposure and outcome. Sensitivity analyses, such as the weighted median^[22]^ and MR-Egger regression,^[23]^ were employed under different assumptions, albeit with reduced statistical power. The weighted median method tolerates up to half of the SNPs being invalid instruments or exhibiting pleiotropy. Consistency across methods was sought for reliability, with divergent results from the 5 methods leading to exclusion. Instrument heterogeneity was evaluated using Cochran *Q*-test,^[24]^ and MR-pleiotropy residuals were utilized for further exclusions, considering a significance level of *P* < .05 to determine statistical significance and evidence of potential causal effects.

### 2.6. Integration of DEGs and positive eQTL findings

The intersection of DEGs and MR-positive eQTL genes was determined using the VennDiagramR package. This process involved intersecting up-regulated DEGs with MR-positive genes exhibiting an effect value >1, and similarly for down-regulated genes. This aggregation identified the key genes associated with RA, from which effect values, 95% confidence intervals, number of SNPs, chromosome numbers, locations, and distances of these genes were derived.

### 2.7. Enrichment analysis of key genes

Upon identifying the key genes, gene ontology enrichment analysis was conducted to categorize these genes according to cellular components, biological processes (BPs), and molecular functions. The KEGG pathway analysis further elucidated the pathways and associated functions of these gene clusters, considering *P* < .05 as statistically significant. Visualization of GO functional enrichment was achieved through bubble, bar, and circle plots, and KEGG pathway enrichment through bubble and bar plots, all generated using respective R packages.

For GSEA single-gene enrichment analysis, the clusterProfiler R package was utilized, selecting the KEGG pathway gene set (c2.cp.kegg.Hs.symbols.gmt) for analysis with key genes displaying the most significant up- and down-regulated effect values. The top 5 pathways were visualized to highlight the GSEA up-regulated and down-regulated enrichment pathways.

### 2.8. Immune cell infiltration assessment

The relative proportions of 22 tumor-infiltrating immune cell subtypes were inferred using the CIBERSORT algorithm applied to batch-corrected GEO chip data.^[25]^ The default LM22 leukocyte gene signature matrix, comprising 547 genes, distinguishes 22 mature human hematopoietic populations, including various T-cell types, B-cells, plasma cells, natural killer (NK) cells, and myeloid subpopulations. Only samples with a CIBERSORT *P* < .05 were considered for further analysis, ensuring each immune cell type score sum equaled 1 per sample. Histograms of immune cell infiltration and correlations between immune cells and key genes were plotted using R packages for visualization.

### 2.9. GEO chip validation and variance analysis

To validate the identified key genes, microarrays from the GEO database were used as a validation group to examine the significant expression differences between key genes across control and experimental groups. The ggpubr R package facilitated the creation of box-and-whisker plots to assess these differences. Genes significantly expressed in the validation group compared to controls were deemed key target genes, reinforcing their potential role in RA pathogenesis and treatment targets.

## 3. Results

### 3.1. Standardized integration of 3 different platform datasets and removal of batch effects

**Figure 2.**
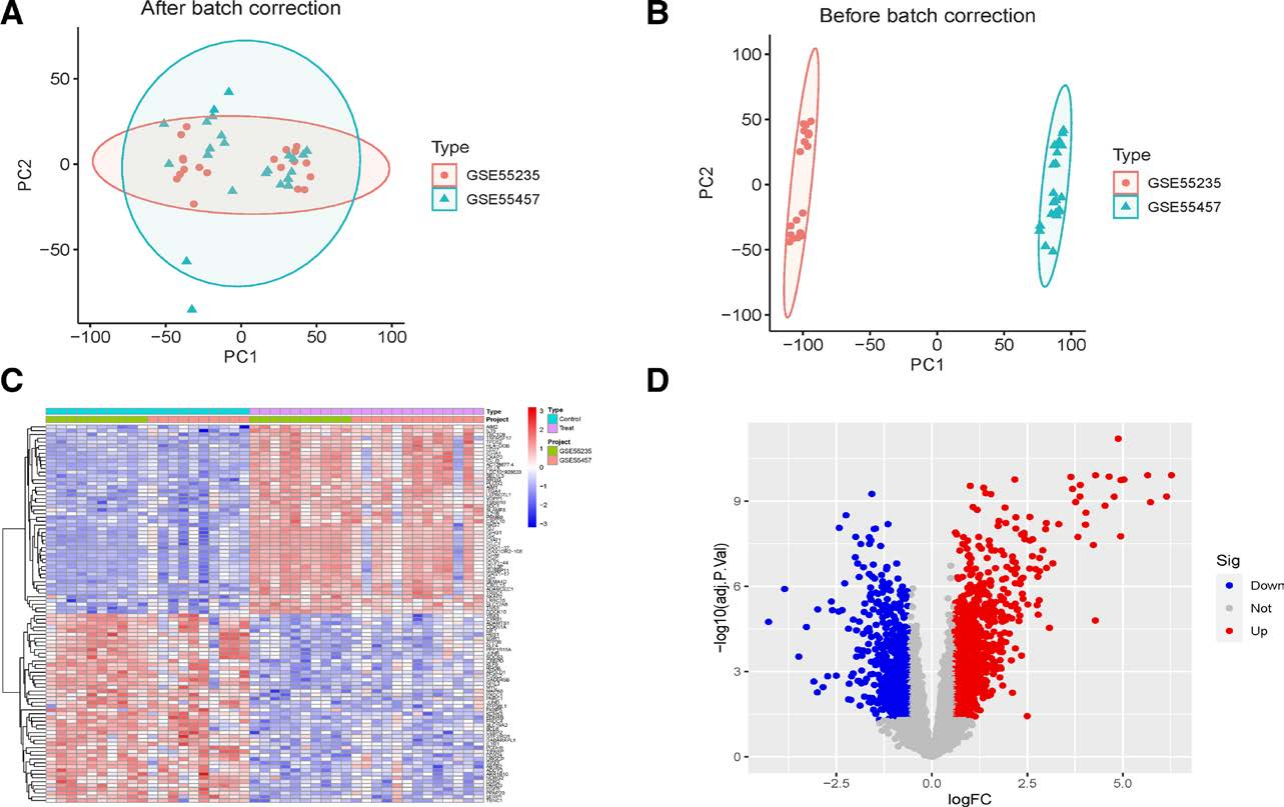
(A) Before batch correction. (B) After batch correction. (C) Heatmap of differentially expressed genes. (D) Volcano plot of differentially expressed genes.

Three RA datasets, GSE55235, GSE55457, and GSE772983, were selected through screening. Each dataset, containing details such as the number of samples and the microarray platform used, underwent a standardized and normalized process for gene expression values. Specifically, the first 2 datasets were designated as the training group, with the latter serving as the validation group. This approach resulted in a compiled dataset of 23 RA patients and 20 healthy controls for the training groups. Batch effects were addressed using the Combat function within the “sva” R package, leading to a unified dataset. Principal component analysis plots, depicted in Fig. 2A and B, illustrate the gene expression patterns before and after batch correction. Initially, the gene expression patterns across different datasets varied significantly, making it challenging to discern the biological differences between the RA and healthy control groups due to pronounced batch effects. Post-correction, the gene expression patterns across datasets appeared consistent, significantly reducing dataset heterogeneity and enhancing the biological distinctions between the RA and healthy control groups. This correction indicated successful mitigation of the batch effects in the integrated datasets, rendering the standardized and integrated dataset suitable for subsequent analyses.

### 3.2. Identification of DEGs

The analysis differentiated between the RA group and the healthy control group within the dataset, encompassing 43 samples and 13,236 genes. The differential analysis revealed 1668 DEGs between the RA and healthy control groups. Of these, 888 genes were up-regulated in the RA group (logFC > 1, *P* < .05), and 780 genes were down-regulated (logFC < 1, *P* < .05). Subsequent analysis of the normalized combined data facilitated the visualization of these DEGs through heatmaps and volcano plots, as shown in Fig. 2C and D. The prioritization of genes in the RRA results was based on the inverse relationship between the *P*-value and gene rank, with lower *P*-values indicating higher confidence in the differential expression. This approach provided a rigorous method for identifying statistically significant genes, ensuring a robust analysis of differential gene expression between the RA and healthy control groups.

### 3.3. Mendelian randomization

MR utilized comprehensive eQTL-associated SNP information from Table S1, Supplemental Digital Content, https://links.lww.com/MD/P663, including β-values, standard errors, effect alleles, and other alleles. The *F*-statistic values for the selected SNPs ranged from 29.71 to 14,552.87, indicating robust IVs without weak instrumental bias. MR analysis, employing 5 methods (IVW, MR Egger, simple mode, weighted median, and weighted mode), investigated causality between eQTL exposures and RA. Inconsistencies across these methods were excluded to ensure reliability of the MR effect sizes. MR polytropic residuals (MR-pleiotropy) and Cochran Q statistic for MR-heterogeneity IVW test were applied, with a significance level of *P* < .05, revealing no significant heterogeneity and confirming the robustness of the results. This rigorous screening yielded 242 eQTLs significantly associated with RA, detailed in Table S2, Supplemental Digital Content, https://links.lww.com/MD/P663.

### 3.4. MR results for key genes

**Figure 3.**
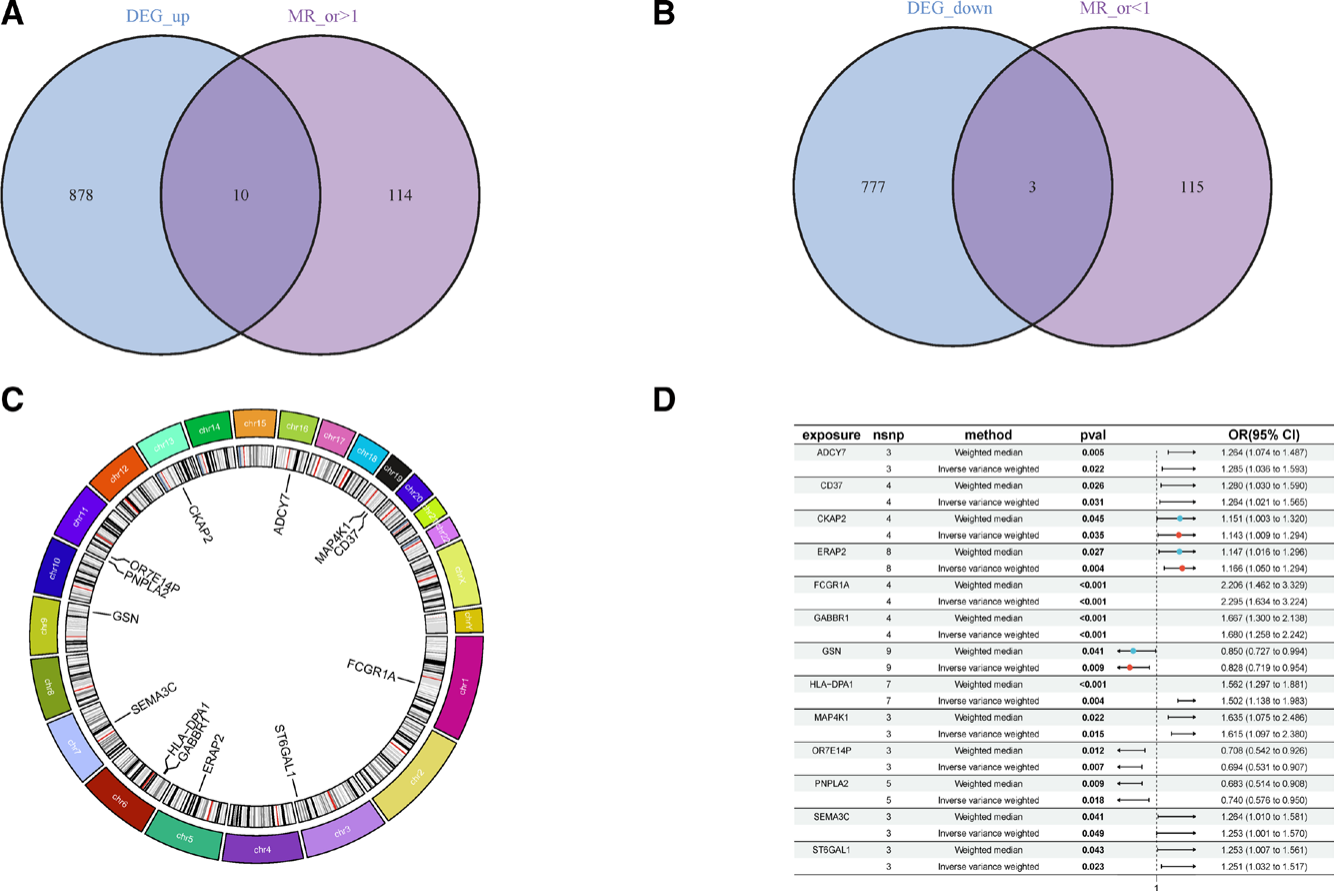
(A) Ten susceptibility genes among the key genes. (B) Three protective genes among the key genes. (C) Chromosomal distribution and location information of the 13 key genes. (D) Mendelian randomization results for the 13 key genes.

**Figure 4.**
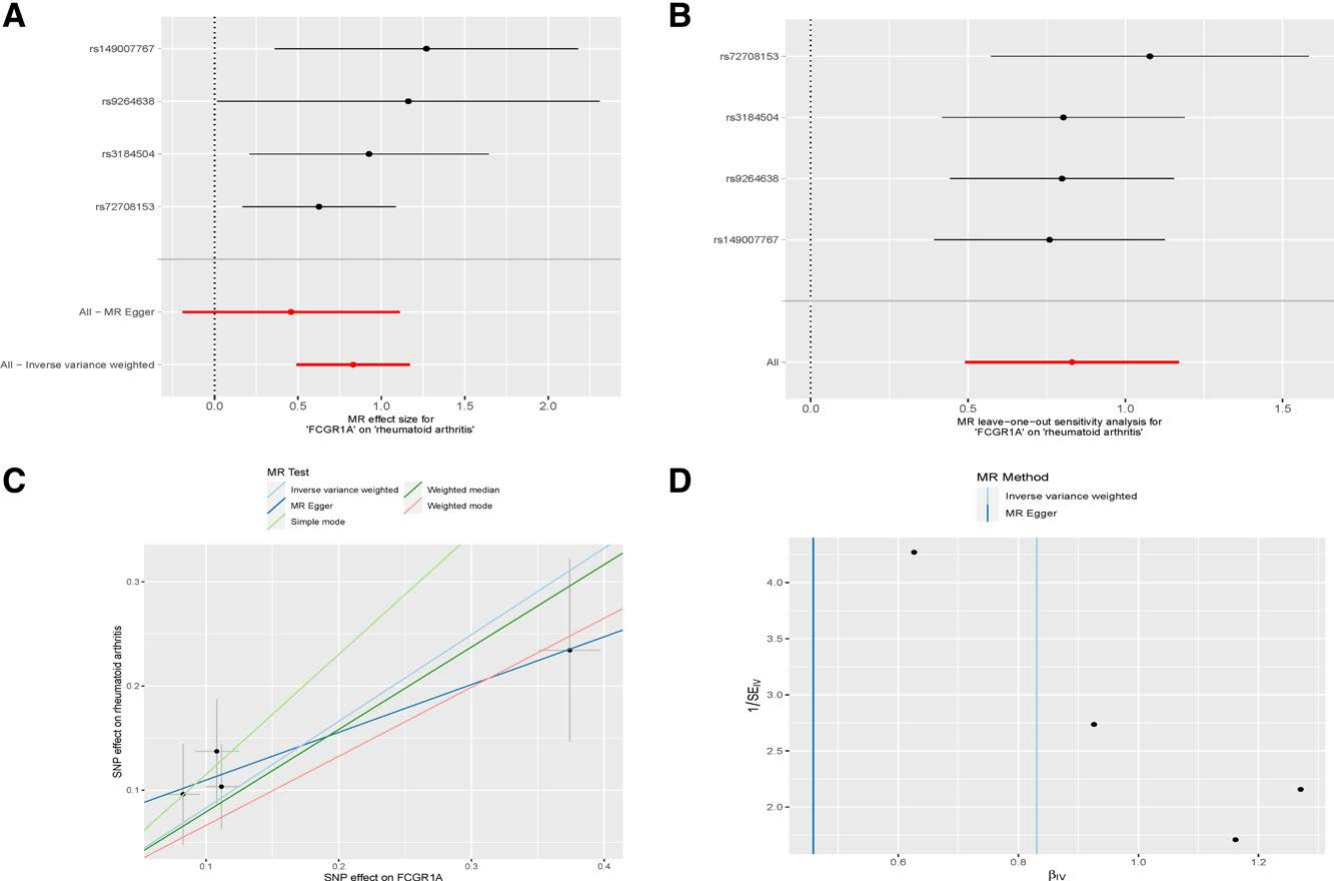
(A) Forest plot of FCG1R versus RA. (B) Leave-one-out analysis of FCG1R versus RA. (C) Scatterplot of FCG1R versus RA. (D) Funnel plot of FCG1R versus. RA = rheumatoid arthritis.

**Figure 5.**
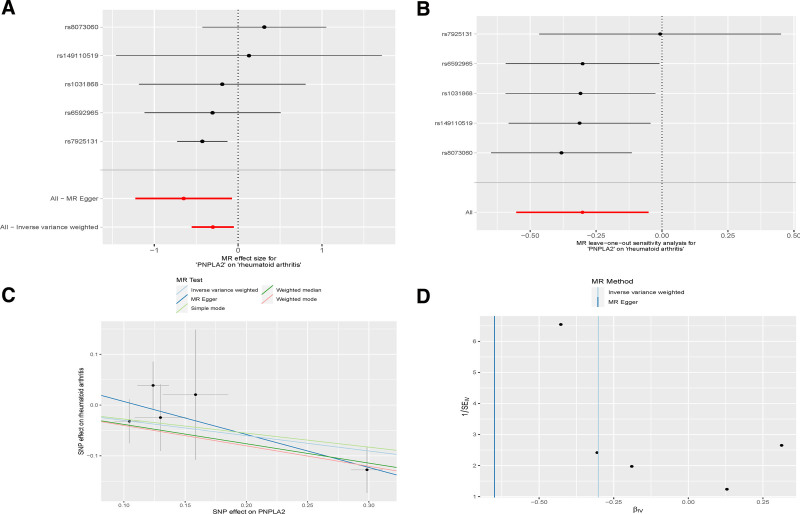
(A) Forest plot of PNPLA2 against RA. (B) Leave-one-out plot of PNPLA2 against RA. (C) Scatterplot of PNPLA2 against RA. (D) Funnel plot of PNPLA2 against RA. RA = rheumatoid arthritis.

The intersection of DEGs with significant GWAS results was determined using the VennDiagram R package. This analysis identified 10 key up-regulated genes with MR Ors >1, suggesting their potential as new susceptibility genes for RA. These genes include CKAP2, GABBR1, HLA-DPA, ST6GAL1, FCGR1A, ADCY7, MAP4K1, CD37, ERAP2, and SEMA3C. Conversely, 3 down-regulated genes with odds ratios <1 (PNPLA2, gelsolin [GSN], and OR7E14P) were identified as potential protective genes against RA. Venn diagrams (Fig. 3A and B) and chromosomal locations of these 13 key genes (Fig. 3C), along with forest plots of MR results (Fig. 3D), underscore their significance. Analysis of multiplicity and sensitivity confirmed no significant pleiotropy, attesting to the robustness of these findings, with specific effect values detailed in Table 1. Notably, FCGR1A emerged as the gene with the highest effect value among up-regulated genes, indicating a significant increase in RA risk with its increased expression, as detailed in Fig. 4. In contrast, OR7E14P was highlighted as the most effective protective gene, with its specific MR analysis shown in Fig. 5.

**Table 1:** Sensitivity analysis of 13 key genes with RA.

Gene	Method	nSNP	Pleiotropy				Heterogeneity	
			MR-PRESSO Global *P*-value	Egger intercept	Intercept’s SE	MR Egger *P*-value	*Q*-value	*P*-value
CKAP2	IVW	4	.437	0.462	0.048	.897	1.901	.593
GABBR1	IVW	4	.961	‐0.004	0.068	.192	5.819	.121
HLA-DPA1	IVW	7	.379	‐0.092	0.095	.055	16.415	.012
ST6GAL1	IVW	3	.659	0.042	0.071	.832	1.171	.557
FCGR1A	IVW	4	.319	0.064	0.049	.301	2.049	.562
ADCY7	IVW	3	.340	0.122	0.072	.945	3.620	.164
MAP4K1	IVW	3	.467	‐0.733	0.660	.413	1.252	.535
CD37	IVW	4	.640	‐0.094	0.172	.394	1.271	.736
ERAP2	IVW	8	.516	0.067	0.097	.805	0.791	.998
SEMA3C	IVW	3	.710	‐0.025	0.050	.354	0.402	.818
PNPLA2	IVW	5	.280	0.072	0.055	.114	3.660	.454
GSN	IVW	9	.756	‐0.009	0.027	.222	5.892	.659
OR7E14P	IVW	3	.900	‐0.009	0.056	.452	1.784	.410

GSN = gelsolin, IVW = inverse variance weighted.

### 3.5. GO and KEGG enrichment analysis of intersecting genes

**Figure 6.**
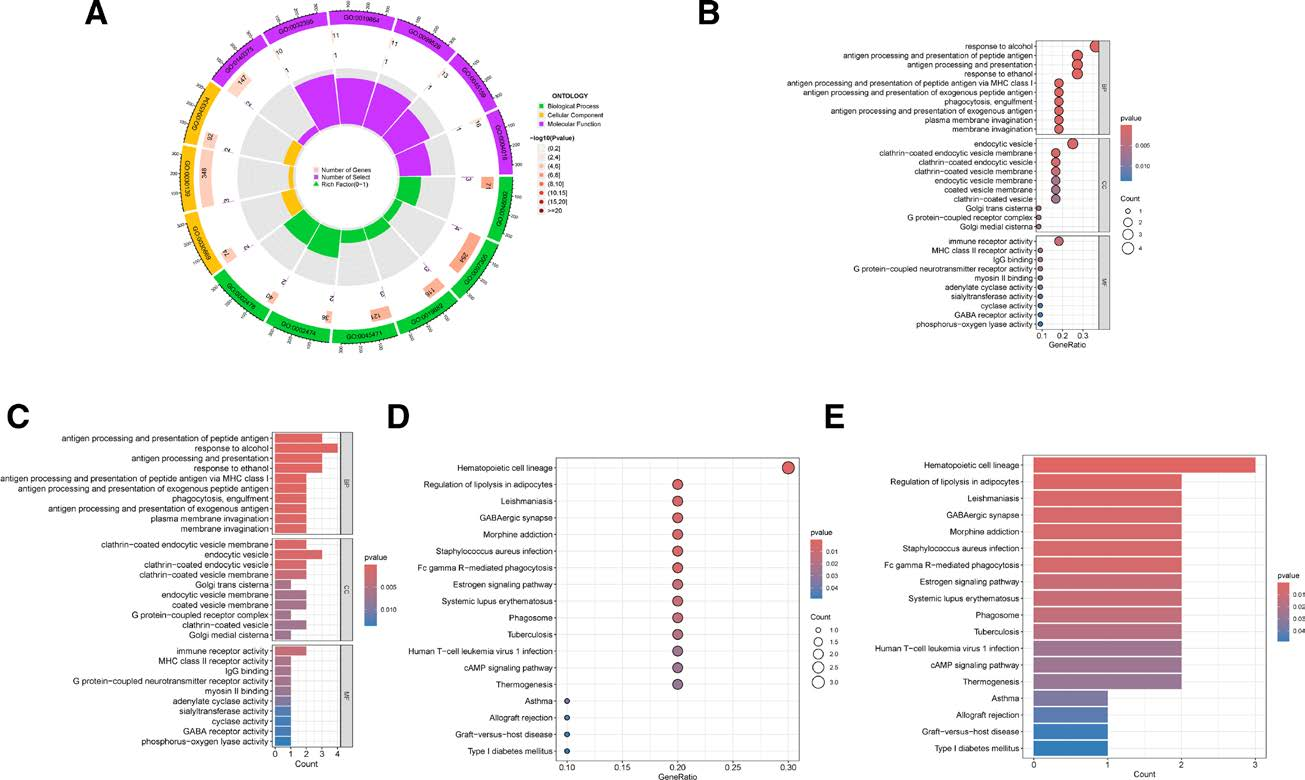
GO and KEGG analysis of key genes. (A) GO enrichment analysis of BP, CC, and MF significant entries. (B) Bubble plots in GO analysis. (C) Bar graph in GO analysis. (D) Bubble plot in KEGG enrichment analysis. (E) Bar graph of KEGG enrichment analysis. BP = biological process, CC = cellular component, GO = gene ontology, KEGG = Kyoto encyclopedia of genes and genomes, MF = molecular function.

This study further investigated 13 key genes associated with RA through GO and KEGG functional enrichment analyses. The GO analysis identified significant enrichments across 13 molecular function categories, with the primary functions including immune receptor activity, major histocompatibility complex (MHC) class II receptor activity, immunoglobulin G (IgG) binding, and G protein-coupled neurotransmitter receptor activity. Additional noteworthy functions involved myosin II binding, adenylate cyclase, glycosyltransferase, cyclooxygenase, GABA receptor activities, and phosphorus-oxygen lyase activity. The analysis also highlighted 16 significantly enriched BP categories, notably involving response to alcohol, antigen processing and presentation of peptide antigens via MHC class I and II, and phagocytosis. Cellular component analysis revealed enrichment in endocytotic vesicles, lectin-encapsulated endocytic vesicle membranes, and aggrecan-coated endocytic vesicles (see Fig. 6A).

KEGG analysis revealed that the RA-associated genes were significantly enriched in 18 pathways, with critical pathways including hematopoietic cell lineage, graft-versus-host disease, allograft rejection, asthma, thermogenesis, cAMP signaling pathway, human T-cell leukemia virus 1 infection, tuberculosis, and systemic lupus erythematosus. Figure 6B and C illustrate the GO enrichments using bubble plots and bar graphs, respectively, while Fig. 6D and E depict the KEGG enrichments. Detailed gene distributions and proportions within each significantly enriched category for both GO and KEGG analyses are provided in Table S3, Supplemental Digital Content, https://links.lww.com/MD/P663 and Table S4, Supplemental Digital Content, https://links.lww.com/MD/P663. This comprehensive analysis underscores the multifaceted biological roles and pathways involved in RA pathogenesis, highlighting the potential of these key genes as therapeutic targets.

### 3.6. Enrichment pathway of key genes analyzed by GSEA

**Figure 7.**
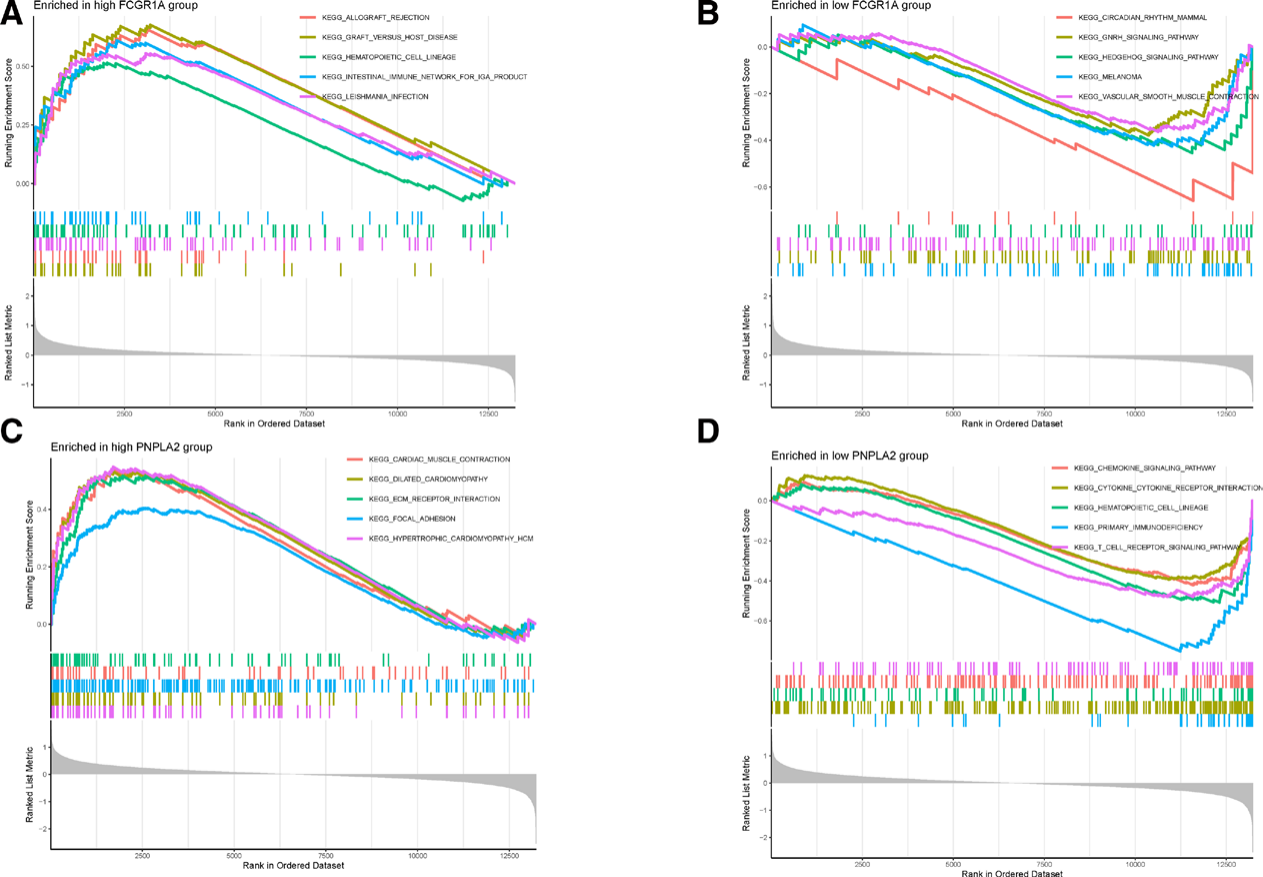
GSEA enrichment analysis. (A) Top 5 significant pathways of the FCG1R gene-enriched high expression group. (B) The first 5 significant pathways of the FCG1R gene-enriched low expression group. (C) Top 5 significant pathways of the PNPLA2 gene-enriched high expression group. (D) Top 5 significant pathways in the PNPLA2 gene-enriched low expression group. GSEA = gene set enrichment analysis.

In the GSEA enrichment analysis of key genes, FCGR1A and PNPLA2 were selected for their significant effect values among the up-regulated and down-regulated genes, respectively. The KEGG pathway enrichment for FCGR1A identified 27 significant pathways. The top 5 enriched pathways for the highly expressed gene set included allograft rejection, graft-versus-host disease, hematopoietic cell lineage, intestinal immune network for IgA production, and leishmaniasis. Conversely, the top 5 pathways for the lowly expressed gene set were mammalian circadian rhythm, GnRH signaling pathway, Hedgehog signaling pathway, melanoma, and vascular smooth muscle contraction. Detailed illustrations of these pathways are shown in Fig. 7A and B. The comprehensive enrichment results can be found in Table S5, Supplemental Digital Content, https://links.lww.com/MD/P663.

For PNPLA2, 39 significant enrichment pathways were identified. The high expression gene set’s top 5 pathways were cardiac muscle contraction, dilated cardiomyopathy, ECM–receptor interaction, focal adhesion, and hypertrophic cardiomyopathy. The low expression gene set’s top 5 pathways included chemokine signaling pathway, cytokine–cytokine receptor interaction, hematopoietic cell lineage, primary immunodeficiency, and T cell receptor signaling pathway. These results are visually represented in Fig. 7C and D. The full list of enrichment pathways is available in Table S6, Supplemental Digital Content, https://links.lww.com/MD/P663.

### 3.7. Immune cell infiltration analysis

**Figure 8.**
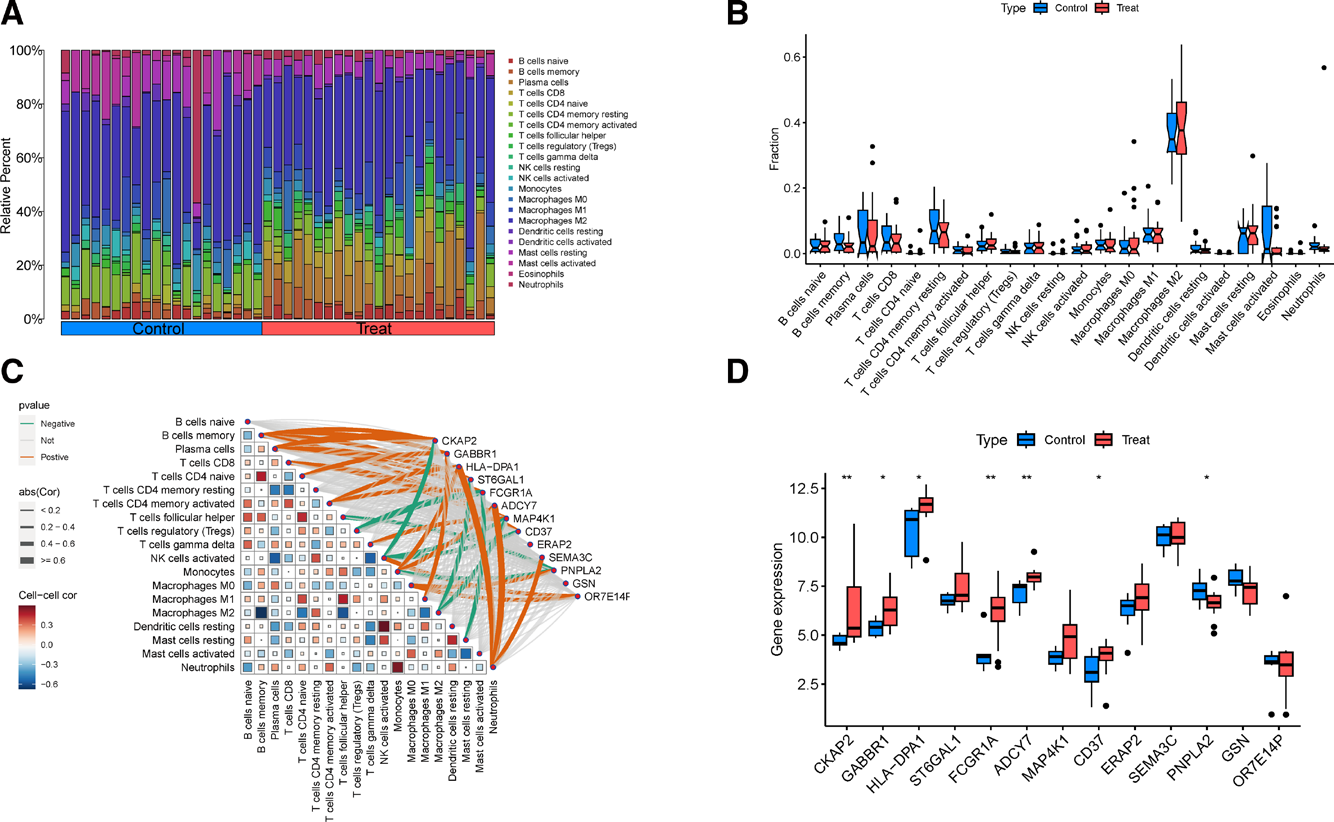
(A) Histogram of immune cell infiltration analysis. (B) Box line plot of analysis of differences between 22 immune cells. (C) Correlation results between 22 immune cells and 13 key genes. (D) External geo data validation of 13 genes with significant expression boxplots.

To further investigate the role of key genes in the pathogenesis and progression of RA, this study conducted an immune cell infiltration analysis using genome-wide expression profiles. Analysis of 22 immune cells revealed that M2 macrophages and CD8+ T cells were predominantly found in RA synovial tissues, as depicted in the bar chart in Fig. 8A. The box-and-whisker plots demonstrated variations in immune cell infiltration between the experimental and control groups across different samples, illustrated in Fig. 8B. Additionally, correlation analysis between the expression of key genes and immune cell abundance (detailed data available in Table S7, Supplemental Digital Content, https://links.lww.com/MD/P663) identified 3 genes (HLA-DPA1, ADCY7, and SEMA3C) as being positively correlated with neutrophils, exhibiting the most significant *P*-values. Similarly, PNPLA2, OR7E14P, and M0 macrophages showed positive correlations. In contrast, ST6GAL1 and MAP4K1 were negatively correlated with M2 macrophages, as presented in Fig. 8C. These findings suggest that genes like HLA-DPA1, ADCY7, and SEMA3C may contribute to the development and progression of RA by modulating neutrophil and macrophage functions.

### 3.8. Validation of key genes in external datasets

The 13 key genes identified through our analysis were subjected to validation using an external dataset, specifically GSE77298, for expression validation. Of these, 7 genes (CKAP2, FCGR1A, ADCY7, GABBR1, HLA-DPA, CD37, and PNPLA2) were successfully validated. Notably, CKAP2, FCGR1A, and ADCY7 demonstrated highly significant associations with *P*-values <.001, denoted by 2 asterisks (**), while GABBR1, HLA-DPA, CD37, and PNPLA2 showed significant associations with *P*-values <.05, marked by one asterisk (*). Some genes, however, were not validated in this dataset. Detailed outcomes are presented in Fig. 8D. These findings suggest that the 7 validated genes could serve as pivotal targets for understanding and potentially treating RA.

## 4. Discussion

RA is predominantly characterized by chronic inflammation of the synovium, leading to the erosion and destruction of joints. Early diagnosis and effective treatment are pivotal in preventing joint damage and enhancing quality of life. However, the early stages of RA are challenging to diagnose due to the absence of effective biomarkers.^[26]^ The discovery of novel and effective biomarkers is essential for the early detection and management of RA. The development of high-throughput microarray technology, coupled with advancements in bioinformatics, enables the identification of potential key genes involved in RA’s pathological mechanisms, offering new avenues for biomarker discovery. The RRA algorithm stands out for its computational efficiency, robustness against background noise, ability to handle incomplete rankings, and provision of significant scores for each element. MR, as a genetic epidemiological approach, addresses the limitations inherent in traditional observational studies by mitigating the impact of various acquired confounders.^[27]^ Our study represents the first to systematically integrate multiple GEO databases with MR, offering novel insights into the pathogenesis of RA.

Previous studies have highlighted CKAP2 as the most significant microvascular growth factor closely linked with the malignant progression of tumor cells.^[28]^ Although literature connecting it to RA is scarce, we have identified neovascularization as a critical characteristic of RA synovitis, marked by extensive synovial infiltration and endothelial cell activation.^[29]^ Furthermore, the expansion of synovial fibroblasts and macrophage-like cells promotes the proliferation of the synovial membrane and invasion of periarticular bone at the cartilaginous junction, leading to bone erosion and cartilage degradation through the NF-κB signaling pathway, an inflammation-related process.^[30]^ This suggests that CKAP2 plays a pivotal role in the pathogenesis of RA. GABBR1, a component of the GABA receptor implicated in inflammation regulation, resides within the MHC extended class I region (6p21.3), influencing diseases like RA.^[31]^ Despite specific HLA-DRB1 alleles (MHC class II) significantly contributing to RA susceptibility, potentially through their role in presenting arthritic peptides, GABBR1 might independently influence genetic susceptibility to RA due to linkage disequilibrium. While direct experimental characterization of GABBR1 in RA patients is pending, computational analysis indicates that polymorphisms could impact alternative splicing or protein structure, with multiple isoforms and missense mutations being closely related to RA.^[32]^ HLA-DPA1, part of the HLA class II α-chain paralogs, forms a heterodimer with α (DPA) and β (DPB) chains, both integral to the immune response by presenting extracellular protein-derived peptides.^[33]^ Previous research has shown HLA-DPB1 as a gene susceptible to RA, while HLA-DPA1, previously associated with conditions like rubella and granulomatous polyangiitis, has been linked to RA in various studies. The strong link between the HLA-DPA1*02:01 allele and RA suggests a higher predisposition to the disease among carriers of this allele. This raises the hypothesis that mutations in these alleles can disorganize the immune system, leading to an erroneous attack on the body’s own tissues, particularly the joints and synovium, thereby intensifying joint inflammation.^[34]^

ST6GAL1, a gene encoding a protein, has been identified through experimental studies to be upregulated by estrogen, impacting IgG secretion. Low IgG secretion levels promote inflammation, whereas high levels can suppress symptoms. The reduction in estrogen, particularly noticeable in postmenopausal women, diminishes IgG secretion, exacerbating inflammation and contributing to the development of RA.^[35]^ This supports the observed epidemiological trend where RA prevalence is higher among women, especially those who are postmenopausal, aligning with our findings. FCGR1A, a high-affinity Fc-γ receptor, plays a crucial role in mediating cytotoxicity and phagocytosis. It balances the immune system’s response to infection and autoimmunity prevention. Disruption of this balance increases susceptibility to autoimmunity and infections.^[36,37]^ In RA, immune complexes formed by autoantibodies such as rheumatoid factor and anti-cyclic citrullinated peptide antibodies activate immune cells through Fcγ receptor interactions, promoting an inflammatory response.^[38]^ ADCY7, a membrane-bound isoform activated by extracellular signals, is pivotal in the catecholamine-stimulated lipolysis pathway. While its role in RA pathogenesis remains underexplored, studies have linked it to synovial inflammation in osteoarthritis. Research by Duan B et al showed that macrophages from ADCY7-deficient mice produced higher levels of the proinflammatory cytokine tumor necrosis factor-α upon stimulation. Suppressing ADCY7 expression mitigated inflammatory lipolysis and fibroblast-like synoviocyte dysfunction in a rat model, suggesting ADCY7 as a potential pharmacological target for treating inflammation in lipid metabolism disorders.^[39]^ Further investigation is required to understand the detailed mechanisms.^[40]^ MAP4K1, also known as hematopoietic progenitor kinase 1, belongs to the mammalian Ste20-like kinase family and is instrumental in immune cell signaling and the inflammatory response.^[41]^ It has been identified as a regulatory gene in autoimmune diseases such as RA and systemic lupus erythematosus, often acting as a susceptibility gene. Overexpression of MAP4K1 correlates with an increased risk of developing RA.^[42]^ CD37, a protein located on the surface of B cells, regulates immune function and plays a significant role in RA pathology.^[43]^ It influences the pathological process by facilitating the production of autoantibodies and promoting inflammatory responses. The upregulation of CD37 in RA may alter B cell signaling, proliferation, and cytokine release, contributing to the disease’s pathogenesis.^[44]^ These findings are corroborated by immune infiltration analysis, highlighting the involvement of various immune cells and pathways, such as T cells, NK cells, antigen presentation, and immunoreceptor activities.^[45]^ Endoplasmic reticulum aminopeptidase 2 (ERAP2), associated with antigen processing and presentation, particularly in the context of MHC class I-mediated presentation, is crucial for cell-mediated immune responses.^[46]^ Dysfunction in ERAP2 increases the risk of developing RA, aligning with our findings on the enriched pathway for MHC class II receptor activity.^[47]^

SEMA3C encodes a neural guidance molecule involved in several BPs, including neural development, cardiovascular formation, and tumor growth.^[48]^ Its direct link to RA, particularly its regulation of the invasive capacity of fibroblast-like synoviocytes, suggests SEMA3C may influence RA’s severity and progression through its role in cell migration and angiogenesis. Further studies are necessary to validate this potential role.^[49]^

PNPLA2, known within the PNPLA family as adipose triglyceride lipase, plays a pivotal role in the initial step of triglyceride lipolysis within adipocytes, transforming triglycerides into diacylglycerol. Research has linked PNPLA2 to conditions such as neutrophilic lipid storage disease with myopathy and primary triglyceride deposition cardiovascular disease, with its actions spanning across pathways like glycerophospholipid biosynthesis and the modulation of insulin-like growth factor transport and uptake through insulin-like growth factor binding proteins. The connection between PNPLA2 and RA, however, remains ambiguous in contemporary studies. According to Patel et al, PNPLA2 may indirectly mitigate the inflammatory response by modulating fatty acid metabolism. Fatty acids and their metabolite, palmitic acid hydroxystearic acids, exhibit anti-inflammatory properties and can modulate the activity of inflammatory cells and the release of inflammatory mediators, potentially alleviating inflammation and joint damage, thereby offering a mechanism to inhibit RA.^[50]^

GSN, an actin-binding protein, plays a crucial role in cell formation, metabolism, and wound healing.^[51]^ Extensive research has highlighted GSN’s protective function in cartilage regeneration and differentiation. Feldt et al conducted a comparative study on macrophage-like synoviocytes and fibroblast-like synoviocytes from patients with knee osteoarthritis and RA against normal subjects. Their findings revealed a significantly lower concentration of GSN in the joints of the experimental groups, corroborating our assertion of GSN’s involvement in actin binding, cell formation, and wound repair. Further supporting our findings is the discovery of a gelsolin-like actin-binding protein, adserverin, in hypertrophic chondrocytes. Overexpression of adserverin in nonhypertrophic chondrocytes leads to the reorganization of the actin cytoskeleton, altering cell morphology through the extracellular signal-regulated kinase 1/2 and p38 mitogen-activated protein kinase pathways. Given these similarities, we hypothesize that GSN operates through a comparable mechanism, offering protection by cytoskeletal remodeling.^[52]^ OR7E14P, part of the extensive family of G protein-coupled receptors and encoded by a single exon gene,^[53]^ has yet to be directly linked to RA in existing research. However, our MR and differential gene analysis have both pointed to a protective role of OR7E14P against RA, marked by significant effect values. This suggests that the relationship between OR7E14P and RA warrants further investigation, potentially revealing the receptor’s therapeutic value as a target in RA treatment. In addition to the genes previously discussed, existing research has underscored that RA is primarily marked by disruptions in the immune system, characterized by the abnormal elevation of auto-reactive CD4+ T cells, pathogenic B cells, M1 macrophages, inflammatory cytokines, chemokines, and autoantibodies in RA patients.^[54]^ The significance of B cells in RA’s pathogenesis has been conclusively demonstrated through the efficacy of B-cell depletion therapies.^[55]^ Furthermore, Fathollahi et al highlighted the critical role of NK cells in RA’s development.^[56]^ These findings indicate a significant proliferation of immune cells within RA’s immune microenvironment, aligning with our immune cell infiltration results.

This study boasts several strengths, notably the integrated use of multi-GEO microarray analysis and the RRA methodology, combined with MR of eQTL genes, to uncover novel biomarkers for RA. It also leverages immune cell infiltration data for a comprehensive examination of RA’s immune microenvironment and utilizes microarray data from external databases to validate our findings. However, several limitations should be considered when interpreting these results. Firstly, the GEO data were sourced from synovial tissues of RA patients, whereas the eQTLs for MR came from blood specimens, introducing some heterogeneity in sequencing depth. Secondly, most eQTLs were represented by a minimum of only 3 IVs per gene after selection, potentially introducing bias, despite meeting the minimum requirements. This could be mitigated in future studies by utilizing larger sample datasets. Lastly, our sample was confined to European populations, necessitating caution when generalizing these findings to other ethnic groups. Thus, further research involving diverse populations is warranted. Despite these limitations, we believe our findings from GEO-binding gene MR offer valuable insights into RA’s pathogenesis. Continued biological experiments are essential to fully decipher RA’s complex biology and clarify its underlying mechanisms.

## 5. Conclusions

In conclusion, our study identified critical genes associated with RA, including CKAP2, GABBR1, HLA-DPA1, ST6GAL1, FCGR1A, ADCY7, MAP4K1, CD37, ERAP2, SEMA3C, PNPLA2, GSN, and OR7E14P, as potential therapeutic targets through RRA analysis of RA molecular characterization, followed by MR of RA’s eQTL data and intersections. This approach has provided deeper insights into the disease’s molecular basis. Furthermore, through GO, KEGG, and GSEA, we established that differential gene enrichment significantly correlates with immune-related pathways, including immune receptor activity and MHC class II receptor activity, which aligns perfectly with the characteristics of RA we investigated.

We also explored the connection between RA and immune cells within the inflammatory microenvironment by analyzing immune cell infiltration of differential genes. This analysis suggested that cells such as M2 macrophages and CD8+ T cells are likely central to the inflammation observed in RA. Our findings were further corroborated by validating 7 genes using additional GEO datasets. However, despite these advancements, the pathomechanism of RA remains incompletely understood, necessitating further research to elucidate the roles of these key genes in the disease’s progression and pathology.

## Acknowledgments

The authors extend their gratitude to the eQTLGen Consortium for the provision of eQTL data, FinnGen for the access to summarized GWAS data, and the GEO database for supplying the gene chip data. These resources have been invaluable to the success of this study.

## Author contributions

**Conceptualization:** Jingtao Zhang.

**Data curation:** Luyao Ma.

**Resources:** Feng Huang.

**Writing – original draft:** Shoujia Shao, Wenxing Zeng.

**Writing – review & editing:** Shoujia Shao, Ziwei Jiang.

## Supplementary Material


